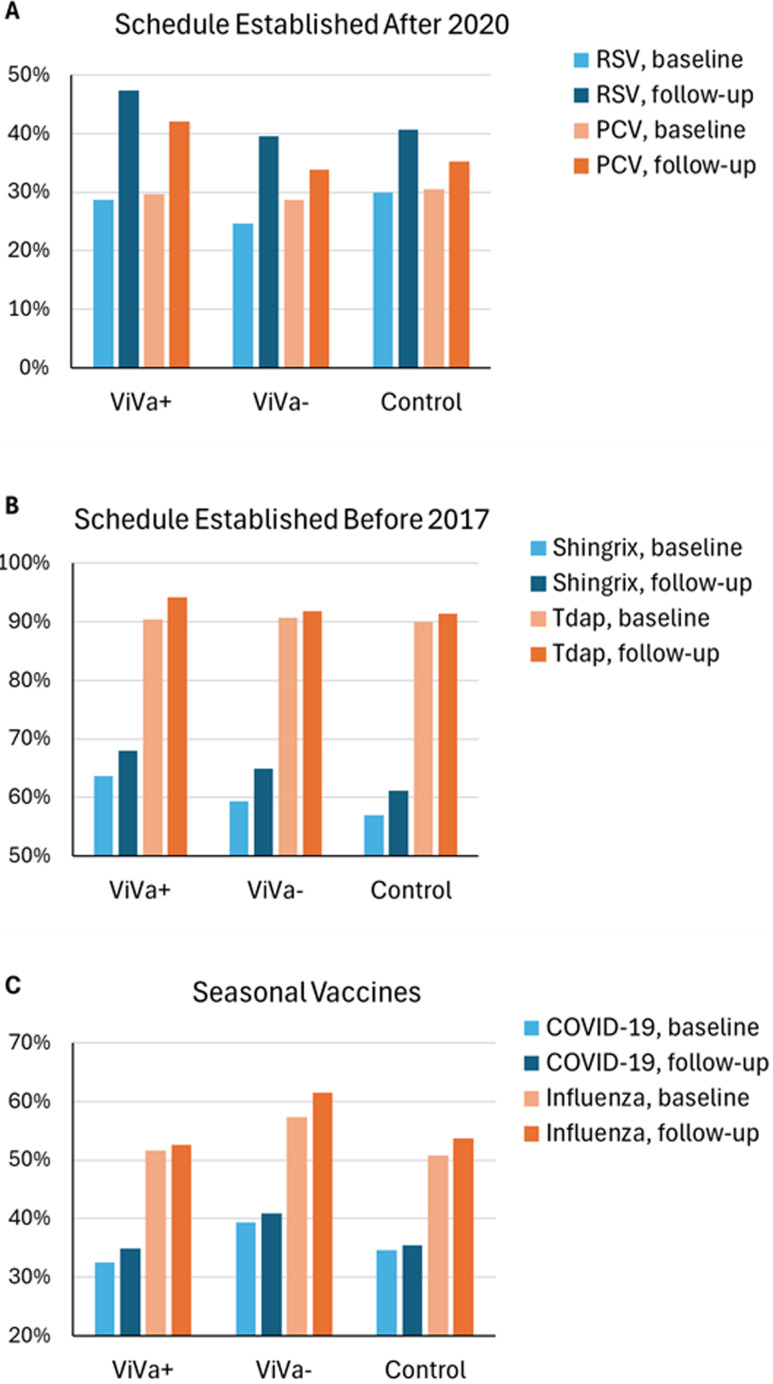# 335 Rising Burden of Carbapenem-Resistant Gram-Negative Pathogens, 1990–2021 to 2050: Implications for Stewardship and Hospital Policy

**DOI:** 10.1017/ash.2026.10738

**Published:** 2026-06-23

**Authors:** Kelly Frank, Brigid Wilson, Taissa Bej, Alison O’Donnell, Robin Jump

**Affiliations:** 1 VA Pittsburgh Healthcare System; 2 Northeast Ohio VA Healthcare System; 3 VA Northeast Ohio Healthcare System; 4 Veteran’s Administration Pittsburgh Health System (VAPHS)

## Abstract

**Background:** Less than 25% of adults in the United States are up-to-date on all age-appropriate vaccinations. Primary care providers (PCPs) may defer routine vaccine discussions due to insufficient time to access and review vaccination records. The Virtual Vaccine (ViVa) clinic was implemented to reduce time and cognitive burden on PCPs by offloading the tasks of vaccine history review and patient-specific recommendations to other clinicians, with the goal to improve adult vaccination rates. **Methods:** From January to October 2025, ViVa clinicians reviewed the charts of Veterans aged ?vs. vs. without (ViVa-) a ViVa note. Patients seen in non-intervention primary care clinics at the same facility served as controls. **Results:** During the study period, 654 and 2488 Veterans aged ?Table 1). The proportion of up-to-date patients increased across all studied vaccines in all groups. Among ViVa+ patients, 36% received one or more recommended vaccines compared to 27% in the ViVa- and 23% in the control patients. The largest gains were for RSV and pneumococcal vaccines among ViVa+ patients (Figure 1). The median number of vaccines for which ViVa+ patients were up-to-date increased from 3 (IQR 2-4) at baseline to 4 (IQR 2-5) at follow-up. Median values did not change from baseline vs. follow-up for patients in the ViVa- (3, IQR 2-4 vs. vs. **Conclusions:** A succinct note entered into the electronic health record successfully augmented adult vaccination rates for patients seen in primary care clinics. The ViVa clinic was more effective for vaccines with recent schedule changes compared to those with more established schedules, including seasonal vaccines. Comparing outcomes among ViVa- and control patients suggests a positive spillover effect for PCPs in the intervention clinics.

**Figure:**
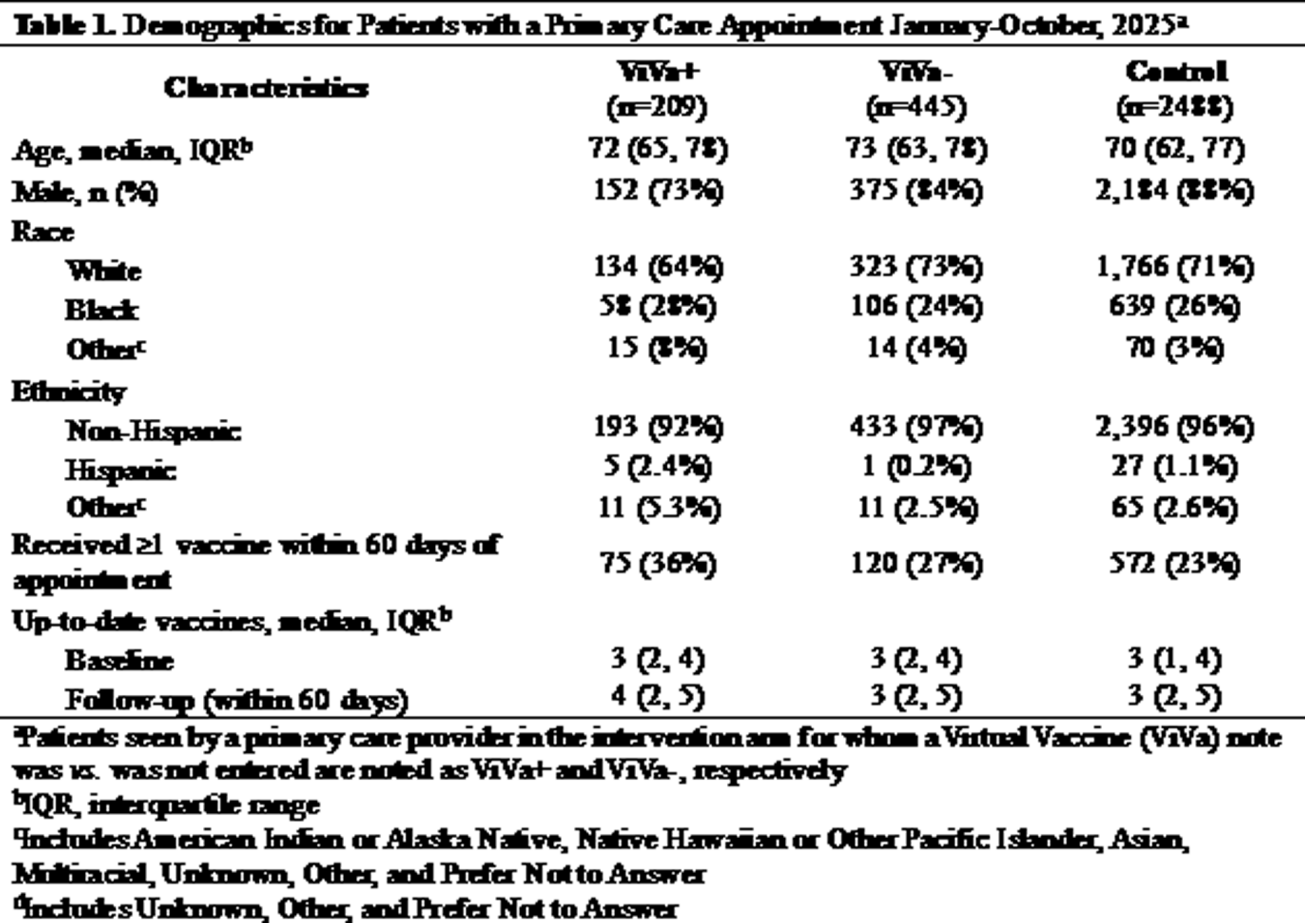

**Figure:**